# Supplementary material for: The Biological Activity of Monarda didyma L. Essential Oil and Its Effect as a Diet Supplement in Mice and Broiler Chicken
Source: Molecules. 2021 Jun 2;26(11):3368. doi: 10.3390/molecules26113368 (PMC8199733; doi:10.3390/molecules26113368)
Supplement: Supplementary file 1 [file molecules-26-03368-s001.zip › molecules-1157792-supplementary.pdf]

molecules

MDPI

Supplementary materials

The biological activity of *Monarda didyma* L. essential oil and its effect as a diet supplement in mice and broiler chicken

Héloïse Côté [1,3], André Pichette [1,3], Alexis St-Gelais [2] and Jean Legault [1,3,*]

[1] Laboratoire d'Analyse et de Séparation des Essences Végétales, Université du Québec à Chicoutimi, 555 boulevard de l'Université, Chicoutimi, QC, G7H 2B1, Canada; heloise.cote@uqac.ca (H.C.); Andre_Pichette@uqac.ca (A.P.); Jean_Legault@uqac.ca (J.L.)

[2] Laboratoire PhytoChemia Inc., 628, Boul. du Saguenay Ouest, Saguenay, QC G7J 1H4, Canada; a.st-gelais@phytochemia.com (A.S.-G.)

[3] Centre de recherche sur la boréalie (CREB), Département des Sciences Fondamentales, Université du Québec à Chicoutimi, 555 boulevard de l'Université, Chicoutimi, QC, Canada, G7H 2B1. ; heloise.cote@uqac.ca (H.C.); Andre_Pichette@uqac.ca (A.P.); Jean_Legault@uqac.ca (J.L.).

* Correspondence: Jean_Legault@uqac.ca

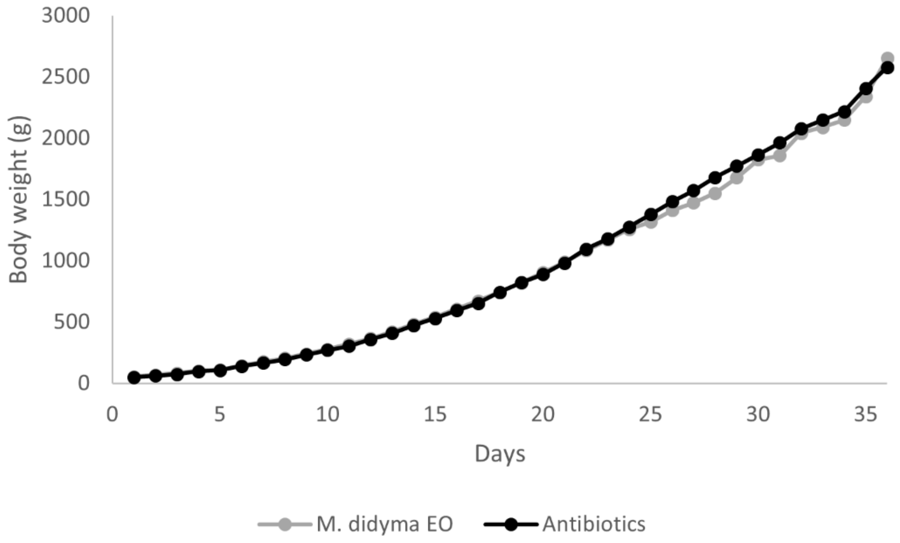

Figure S1. Body weight gain for *M. didyma* essential oil and antibiotics groups for day 0-36. Data represent de mean of the two groups.

Table S1. Composition of *Thymus vulgaris* L. essential oil

RI	Identified compounds<br>Name	Identification[1]	Relative concentration (%)
775	Methyl 2-methylbutyrate	MS, RI	0.31
927	α-Thujene	MS, RI	0.61
932	α-Pinene	MS, RI	1.58
944	Camphene + α-Fenchene	MS, RI	1.83
972	β-Pinene + Sabinene	MS, RI	0.31
983	Octen-3-ol	MS, RI	1.36
988	Octan-3-one	MS, RI	0.12
994	Myrcene	MS, RI	1.22
999	Octan-3-ol	MS, RI	0.26
1003	α-Phellandrene + Pseudolimonene	MS, RI	0.14

1016	α-Terpinene	MS, RI	1.12
1027	*para*-Cymene	MS, RI	43.12
1028	Limonene + β-Phellandrene + 1,8-Cineole	MS, RI	1.22
1059	γ-Terpinene	MS, RI	8.16
1065	*cis*-Sabinene hydrate	MS, RI	0.57
1087	Terpinolene + *para*-Cymenene + *trans*-Linalool oxide (furanoid)	MS, RI	0.30
1096	*trans*-Sabinene hydrate + 6,7-Epoxymyrcene	MS, RI	0.31
1103	Linalool	MS, RI	4.17
1118	*cis-para*-Menth-2-en-1-ol	MS, RI	0.13
1137	Camphor + *trans-para*-Menth-2-en-1-ol	MS, RI	1.37
1162	Borneol	MS, RI	2.79
1174	Terpinen-4-ol	MS, RI	1.28
1182	*para*-Cymen-8-ol	MS, RI	0.16
1187	α-Terpineol	MS, RI	0.26
1192	*cis*-Piperitol	MS, RI	0.11
1235	Thymol methyl ether + Cuminal	MS, RI	0.22
1243	Carvacrol methyl ether	MS, RI	0.61
1259	Geraniol	MS, RI	0.10
1286	Bornyl acetate	MS, RI	0.53
1290	Isothymol	MS, RI	0.33
1299	Thymol	MS, RI	15.38
1300	Thymol isomer	MS, RI	0.15
1305	Carvacrol	MS, RI	1.44
1374	Bornyl propionate	MS, RI	0.10
1380	β-Bourbonene	MS, RI	0.10
1414	β-Caryophyllene	MS, RI	2.32
1440	Unknown [m/z 151, 166 (42), 133 (18), 105 (17), 152 (10)]	MS, RI	0.16
1473	γ-Muurolene	MS, RI	0.12
1477	Geranyl propionate	MS, RI	0.12
1509	γ-Cadinene	MS, RI	0.37
1516	*trans*-Calamenene	MS, RI	0.13
1520	δ-Cadinene	MS, RI	0.16
1574	Caryophyllene oxide	MS, RI	1.10
	Total		96.25

[1]MS: Identification by GC-MS; RI: Compounds were identified by comparison of GC retention indices relative to retention times of a series of n-alkanes(C7-C36) and compared with literature data. Compounds ≤ 0.1 % are not reported.

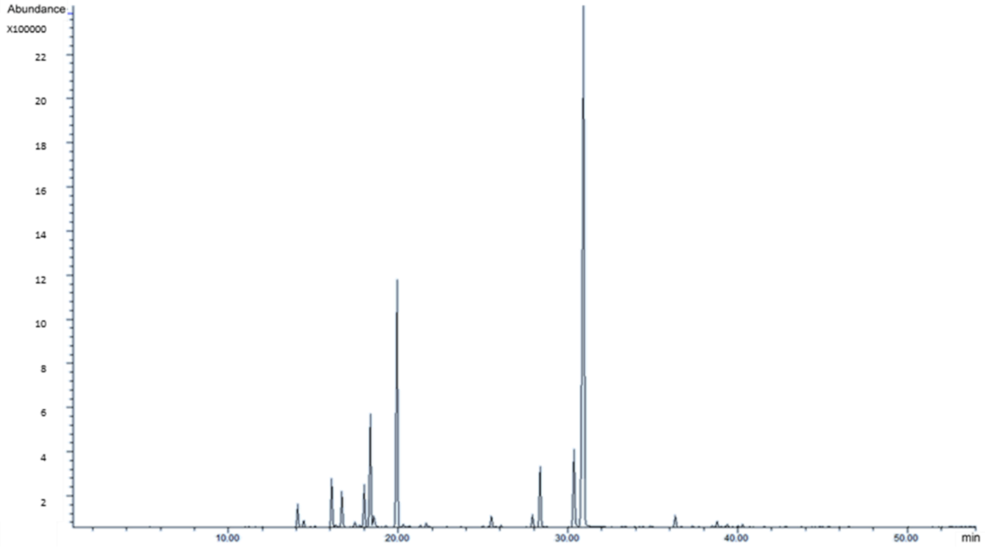

Figure S2. Chromatograms with GC-FID obtain for *M. didyma* essential oil
